# Role of Dicer as a prognostic predictor for survival in cancer patients: a systematic review with a meta-analysis

**DOI:** 10.18632/oncotarget.12183

**Published:** 2016-09-21

**Authors:** Wanying Shan, Chaoyang Sun, Bo Zhou, Ensong Guo, Hao Lu, Meng Xia, Kezhen Li, Danhui Weng, Xingguang Lin, Li Meng, Ding Ma, Gang Chen

**Affiliations:** ^1^ Cancer Biology Medical Centre, Tongji Hospital, Tongji Medical College, Huazhong University of Science and Technology, Wuhan, Hubei, P.R.China

**Keywords:** Dicer, cancer, prognosis, hazard ratio, meta-analysis

## Abstract

**Objective:**

The role of Dicer in the prognosis of cancer patients remains controversial. This systematic review is attempted to assess the influence of Dicer as a prognostic predictor for survival in diverse types of cancers.

**Methods:**

Studies were selected as candidates if they published an independent evaluation of Dicer expression level together with the correlation with prognosis in cancers. Random-effect model was applied in this meta-analysis. Heterogeneity between studies was assessed by Q-statistic with *P* < 0.10 to be statistically significant. Publication bias was investigated using funnel plot and test with Begg's and Egger's test. *P* < 0.05 was regarded as statistically significant.

**Results:**

24 of 44 articles revealed low Dicer status as a predictor of poor prognosis. The aggregate result of overall survival (OS) indicated that low Dicer expression level resulted in poor clinical outcomes, and subgroup of IHC and RT-PCR method both revealed the same result. Overall analysis of progression-free survival (PFS) showed the same result as OS, and both the two subgroups divided by laboratory method revealed positive results. Subgroup analysis by tumor types showed low dicer levels were associated with poor prognosis in ovarian cancer (HR = 1.93, 95% CI: 1.19-3.15), otorhinolaryngological tumors (HR = 2.39, 95% CI: 1.70-3.36), hematological malignancies (HR = 2.45, 95% CI: 1.69-3.56) and neuroblastoma (HR = 4.03, 95% CI: 1.91-8.50).

**Conclusion:**

Low Dicer status was associated with poor prognosis in ovarian cancer, otorhinolaryngological tumors and ematological malignancies. More homogeneous studies with high quality are needed to further confirm our conclusion and make Dicer a useful parameter in clinical application.

## INTRODUCTION

microRNAs (miRNAs) are evolutionarily conserved small RNA molecules, which are predicted to regulate protein synthesis in more than 60% of human genes [1]. By silencing tumor suppressive and oncogenic mRNAs, miRNAs themselves can function as oncogenes or tumor suppressors, respectively [2]. miRNAs play an important role in almost all tumor malignant phenotype, including cancer proliferation, invasion, migration, etc. Considering the broad functional involvement of miRNAs in tumor progression, it is not surprising that miRNAs expressions are associated with cancer prognosis [3, 4].

Dicer(a component of Ribonuclease III), which was firstly reported in 2001, is a cytoplasmic RNase III type endonuclease that specially cleave double-stranded RNAs and an essential protein component of the microRNA (miRNA) machinery with a key function in the maturation of miRNAs [5]. Primer studies have described a global down regulation of miRNAs in cancer [3, 6, 7]. Since Dicer is one of the most important components in miRNA biogenesis process, its expression level seemed to exist some relationships with tumor initiation and progression and patients' prognosis. In recent years, an increasing number of studies have reported the effects of Dicer expression level on patients' prognosis in different types of tumors [8], but still generated conflicting results. Down-regulation of Dicer was reported in some studies to be associated with poor prognosis of cancer, like ovarian carcinomas [9], colorectal cancers [10], breast cancers [11] and etc., while some opposite results also exist [12-14].

In this study, we performed a systematic review of the effects of Dicer on prognosis in all kinds of cancers that had been reported up to Apir 15, 2016, with a following meta-analysis to further assess the influence of Dicer on overall survival (OS) and progression free survival (PFS) in cancer patients.

## RESULTS

### Study selection and characteristics

**Figure 1:**
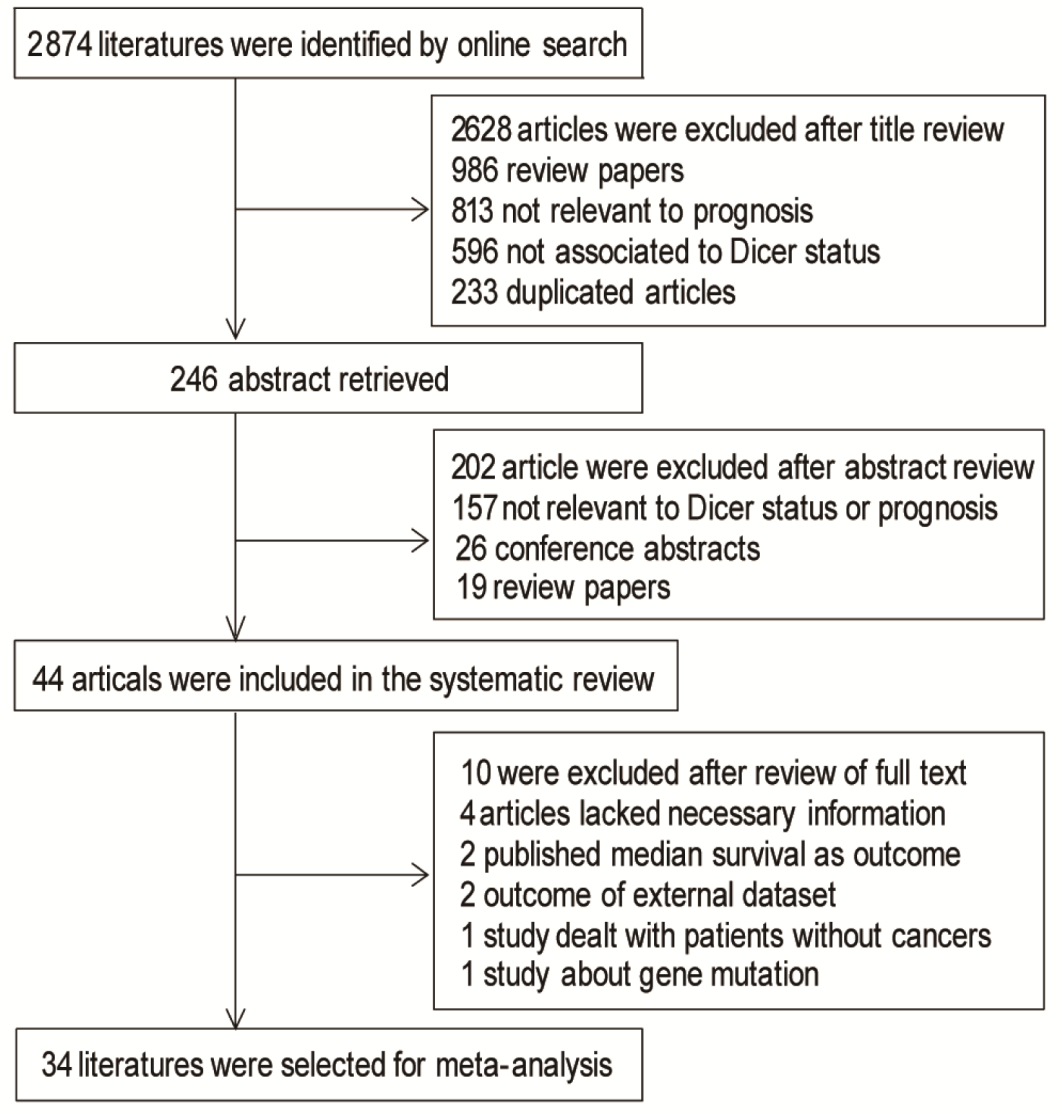
Flow chart of publication selection

In the primary search of the online database, we got 2,874 publications in total, 2,628 of which were excluded after screening the titles. Abstracts of the remaining 246 publications were reviewed and 202 of them were excluded. The remaining 44 publications (Apir 15, 2016), which were completely case-control studies, were screening out as eligible for systematic review. Among the 44 literatures, there were 6 articles studying on breast cancer [11, 14-17], 5 literature about colorectal cancer [10, 12, 18-20], 7 researches on other digestive cancer [21-27], 4 studies on ovarian cancer [9, 13, 28, 29] and 2 on other gynecology cancer [30, 31]. ENT tumors were analyzed in 5 publications [32-36], while hematological cancer such as AML or CLL were describe in 7 articles [37-43], together with 3 papers dealing with prognosis of lung cancer [44-46], 2 studies about neuroblastoma [47, 48],1 research on soft tissue sarcoma [49], 1 study on melanoma cell invasion [50] and 1 study on bladder carcinoma [51]. We reviewed all of the 44 publications in detail, and finally excluded 10 articles from meta-analysis as follows: 4 articles lacked necessary information [23, 25, 32, 50], 2 outcome of external dataset, 1 study only reporting outcome of patients without cancers, 1 study about gene mutation and 2 study reporting the outcome of patients in median survival [17, 18, 20, 41, 42], as shown in Figure 1. Three investigators reviewed all of the 44 candidate articles independently and got an agreement on the exclusion. Among all kinds of these tumors, twenty-four (24/34, 70.59%) articles showed a positive relationship between low Dicer level and poor prognosis results, meanwhile, ten (10/34, 29.41%) studies revealed a negative outcome. Global quality assessment score of the articles included in meta-analysis ranged from 50% to 93.75%, with a median of 76.12%. Detail features of these studies were shown in S1_Table 1 in supplementary file.

Immunochemistry (IHC) method was applied in twenty-one (21/34, 61.76%) studies to detect the expression level of Dicer (S1_Table 2). Monoclonal mouse anti-Dicer antibody was used in thirteen (13/21, 61.90%) studies, while polyclonal rabbit anti-Dicer antibody was used in two (2/21, 9.52%) articles and no full information about antibody in seven (7/21, 33.33%) studies (one trial used both monoclonal and polyclonal antibody). Antigen retrieval was performed in at least fifteen (15/21, 71.43%) literatures while not mentioned in other six (6/21, 28.57%) studies. All of the 21 studies evaluated Dicer levels by scoring the staining intensity although the scoring criterion may be a little different. The summary proportion of low Dicer level in all of the 21 studies was 50.77%. Fourteen (14/21, 66.67%) literatures revealed positive results that low Dicer level was related to poor prognosis in cancer patients. However, the other seven (7/21, 33.33%) articles showed conflicting consequences.

Fourteen (14/34, 41.18%) studies assessed mRNA level of Dicer by means of RT-PCR (S1_Table 3). Mean proportion of low Dicer mRNA level in summary was 46.83%. Among the 14 articles, eleven (11/14, 78.57%) literatures identified low mRNA level as a predictor for poor prognosis.

### Meta-analysis of Dicer expression level and OS

Random effect model was applied to quantitative aggregation of the survival data in this meta-analysis, since the discordance between some studies was obvious. The overall meta-analysis of OS included 26 aggregative studies with 3874 patients (1,939/1,935), among which 17 studies with 2772 patients (1,403/1,369) using IHC method to measure Dicer status and 10 trials with 1,204 patients (588/616) gained mRNA level of Dicer through RT-PCR (one article [30] with 102 patients (52/50) reported both IHC and RT-PCR results, and we selected data of IHC method in the meta-analysis).

Meta-analysis of the 26 literatures was performed and subgroups were divided by different laboratory methods as IHC (1,403/1,369) and PCR (588/616) method. The overall heterogeneity in this analysis was high (I^2^ = 75.4%, *P* = 0.000) while the heterogeneity in IHC and PCR subgroups were also quite obvious (I^2^ = 78.1%, *P* = 0.000; I^2^ = 71.2%, *P* = 0.001). Low Dicer expression was associated with poor OS in the overall analysis with HR = 1.53, 95% CI: 1.19-1.98 and Z = 3.29, *P* = 0.001. In the subgroup analysis according to different laboratory methods, subgroup using IHC method (HR = 1.43, 95% CI: 1.01-2.43) and PCR method (HR = 1.77, 95% CI: 1.14-2.76) both showed a positive result that low Dicer expression level reveled poor OS. Significance tests for IHC subgroup and RT-PCR subgroup were Z = 2.03, *P* = 0.042; Z = 2.55, *P* = 0.011, respectively.

### Meta-analysis of Dicer expression level and PFS

Overall analyses of PFS included 24 articles with 4,523 patients in total. Among the 24 articles, 13 trails with 3268 patients (1,784/1,484) assessed Dicer level with IHC method and 12 studies of 1,357 patients (668/689) with RT-PCR method (one trial [30] with 102 patients measured Dicer level with both methods, we took data of IHC method in the analysis). Since the heterogeneity was noticeable in both analyses, we applied random effect model to analyze the survival data.

**Figure 2:**
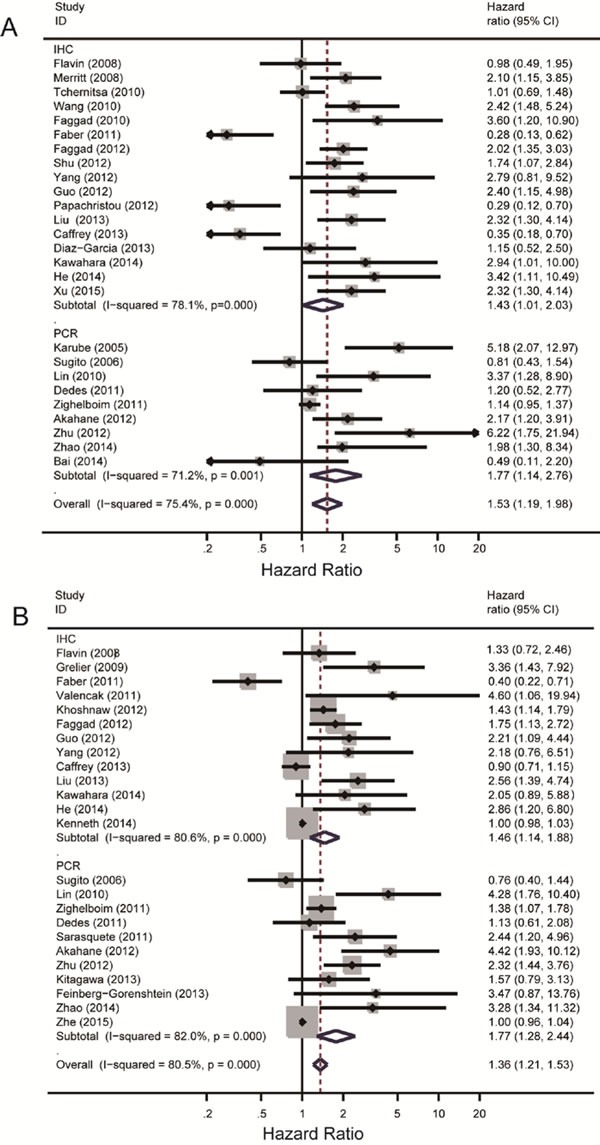
Summary hazard ratios and 95% confidence intervals (CIs) of cancer patients Horizontal lines represent 95% CIs; diamonds represent summary estimates with corresponding 95% CIs. A random-effects model was used for analysis. **A.** For OS group, test for heterogeneity: I^2^ = 78.1%, *P* = 0.000. A random-effects model was used for analysis. **B.** For PFS group, test for heterogeneity: I^2^ = 71.2%, *P* = 0.001.

High heterogeneity existed in the overall analysis (*I*^2^ = 80.5%, *P* = 0.000). Subgroup analysis was carried out as mentioned before and the two subgroups were named as IHC (1,784/1,484) and PCR (668/689). Both heterogeneity of IHC (I^2^ = 80.6%, *P* = 0.000) and RT-PCR (I^2^ = 82.0%, *P* = 0.000) subgroup was statistically significant. The overall effect value showed a significant relationship between low Dicer expression level and decreased PFS with HR = 1.36, 95% CI: 1.21-1.53. Subgroup analysis revealed the same correlation with HR = 1.46, 95% CI: 1.14-1.88 in IHC subgroup (Figure 2A) and HR = 1.77, 95% CI: 1.28-2.44 in RT-PCR subgroup (Figure 2B). Significance tests for overall analysis, results of IHC subgroup and RT-PCR subgroup were Z = 5.11, *P* = 0.000; Z = 2.9, *P* = 0.003; Z = 3.46, *P* = 0.001 respectively, suggesting the significance of results. Although the heterogeneity was evident, meta-regression still showed no probable factors as the main source of it.

### Sensitivity analysis

**Figure 3:**
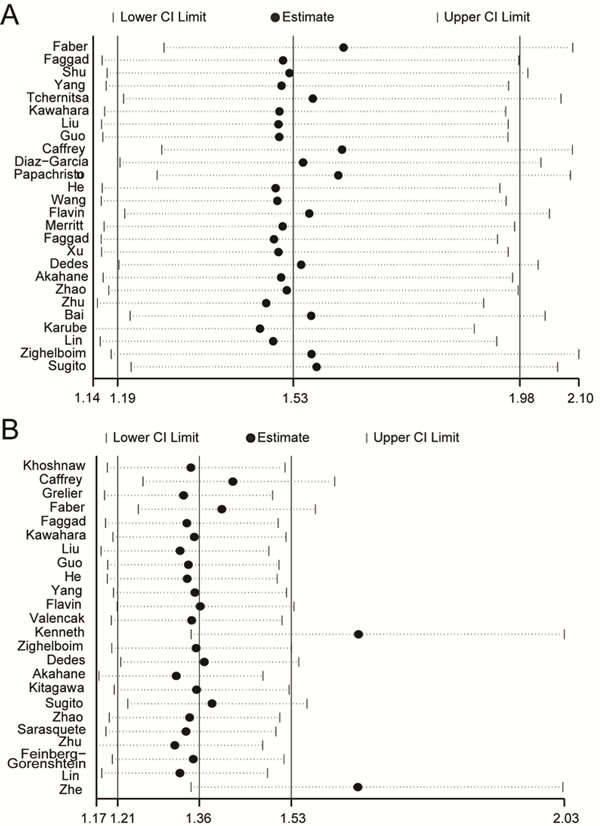
Sensitivity analysis Pooled relative risk and 95% CIs by omitting each study. **A.** For OS group. **B.** For PFS group.

To evaluate the stability of the analysis results above, sensitivity analysis was performed. By removing one study at one time, the overall effect size of the remaining studies was calculated to confirm the effect of the removed one on the whole analysis. As showed in Figure 3, our analysis results were proven to be stable in OS group, but there were two studies [46, 51] impact on the whole stability of PFS group (Figure 3). We excluded the two studies and did meta-analysis again, finally find the heterogeneity decreased from80.5% to 72.4%. (S1_Figure 1)

### Publication bias

**Figure 4:**
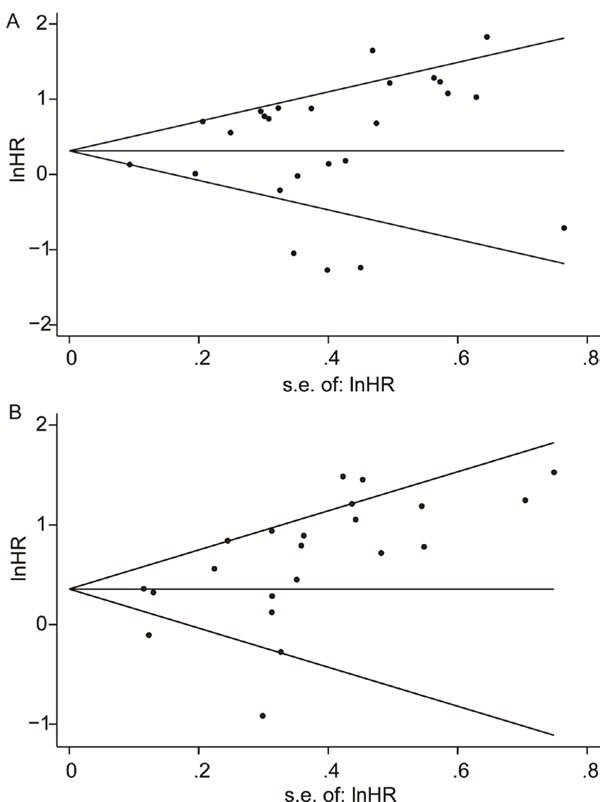
Funnel plots of the hazard ratios of cancer patients by the SE, for all studies included in the meta-analyses Hazard ratios are displayed on a logarithmic scale. **A.** For the OS group, *P* = 0.708 for Begg's test, and *P* = 0.216 for Egger's test. **B.** For the PFS group, *P* = 0.309 for Begg's test, and *P* = 0.000 for Egger's test.

Publication bias was performed using Begg's linear regression test. There was no publication bias in the meta-analysis of studies that deal with OS of patients (Begg's test, *P* = 0.708; Egger's test, *P* = 0.216). However, PFS group was illustrated exist publication bias in the meta-analysis (Begg's test, *P* = 0.028, Egger's test, *P* = 0.011) (Figure 4). The bias may came from the limitation of published studies and positive results were easier to be published due to the mechanism of Dicer.

### Meta-analysis of Dicer level in some types of tumors

**Figure 5:**
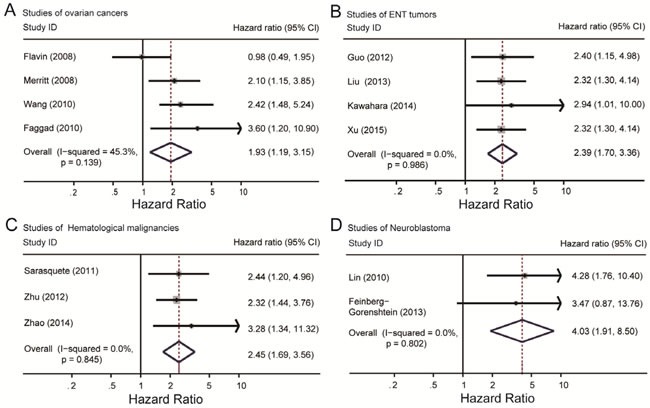
Summary hazard ratios and 95% confidence intervals (CIs) of cancer patients Horizontal lines represent 95% CIs; diamonds represent summary estimates with corresponding 95% CIs. **A.** For ovarian cancer subgroup, I^2^ = 45.3%, *P* = 0.139. **B.** For ENT tumor subgroup, I^2^ = 0.0%, *P* = 0.986. **C.** For neuroblastoma subgroup, I^2^ = 0.0%, *P* = 0.802. **D.** For hematological malignancy subgroup, I^2^ = 0.0%, *P* = 0.845

Since there were several types of tumors included in this study, we intended to find out whether the effect of Dicer level on prognosis was significant in a certain type of tumor. Subgroup analysis according to tumor types was performed and finally found out positive results in ovarian cancer (198/220) and otorhinolaryngological tumor (ENT tumor) (443/316) in the OS group, together with hematological malignancy (312 in totals) and neuroblastoma (44/68) in PFS group. The heterogeneity of ovarian cancer was I^2^ = 45.3%, *P* = 0.139, and combine result of HR was HR = 1.93, 95% CI: 1.19-3.15 (Figure 5A). In ENT tumor subgroup, heterogeneity and HR were *I*^2^ = 0.0%, *P* = 0.986 and HR = 2.39, 95% CI: 1.70-3.36 (Figure 5B). Results of hematological malignancy and neuroblastoma were I^2^ = 0.0%, *P* = 0.845 with HR = 2.45, 95% CI: 1.69-3.56 and I^2^ = 0.0%, *P* = 0.802 with HR = 4.03, 95% CI: 1.91-8.50 respectively (Figure 5C, 5D). The results indicated the absence of obvious heterogeneity in these four subgroups. Entire combine HRs were statistically significant and showed a positive association between low Dicer level and poor prognosis of cancer patients. However, studies on breast cancer (I^2^ = 85.0%, *P* = 0.001) and digestive system cancers (I^2^ = 86.5%, *P* = 0.000) showed obvious heterogeneity and we can't get any significant results. Detail information was listed in Table 1.

**Table 1:** Meta-analysis results of subgroups

subgroup	number of studies	number of patients	ES[95% Conf. Interval]		Test of Heterogeneity	Significance test(s) publication bias				
			HR	95%CI	I-squared		Z	*P*	begg's *P*	egger's *P*
ovarian cancer	4	418	1.93	1.19 to 3.15	45.30%		2.65	0.008	0.734	0.679
neuroblastoma	2	112	4.03	1.91 to 8.50	0.00%		3.65	0	-	-
ENT tumor	4	759	2.39	1.70 to 3.36	0.00%		5.00	0	0.174	0.017
hematological malignancy	3	312	2.45	1.69 to 3.56	0.00%		4.72	0	0.296	0.213
breast cancer	3	1678	1.4	0.85 to 2.30	85.00%		1.32	0.186	1	0.584
digestive system cancers	5	865	1.09	0.49 to2.41	86.50%		0.21	0.836	1	0.777

HR: hazard ratio. 95% CI: 95% confidence interval.

## DISCUSSION

MicroRNAs (miRNAs) are small regulatory RNAs that play an important role in cancer by regulating the expression of a vast number of target messenger RNAs. Dicer is one of the key enzymes involved in the biogenesis and maturation of miRNAs. miRNA biosynthesis is essential for basic cellular functions, such as stemness, cell-cycle progression, mitosis, organogenesis, apoptosis, cell differentiation and proliferation which are ostensibly essential at all stages of tumorigenesis [17, 19]. Several studies have described a trend of down regulation of miRNAs in cancer [3, 6, 7]. These results extend earlier clinical findings, demonstrating that miRNA levels are frequently reduced in cancers, and low levels of Dicer are associated with cancer invasion, distant recurrence and poor overall survival in a variety of cancers [1, 13, 29]. As revealed by knockout of Dicer in mice model of prostate cancer, the migratory capacities of some prostate cancer cell lines were enhanced obviously. Hemizygous loss of Dicer reduces primary tumor burden, but induces a more locally invasive phenotype and causes seminal vesicle obstruction at high penetrance [52].

Although Dicer downregulation in cancer has been reported to be associated with poor prognosis which was proved by our study, the mechanism is not fully understood. By now, it is not clear whether loss of one or more specific miRNAs underlies this effect. It has been reported that Dicer downregulation was involved in the process of tumor proliferation, invasion and metastasis through tumor specific suppressing miRNAs, such as miR-141 in colorectal cancer [18], let-7, miR-128b, and miR-200c in non-small cell lung cancer [44, 53], miR-125a or miR-125b in breast cancer [54], and miR-7 in glioma [55]. Moreover, the specific mechanisms behind Dicer downregulation in cancers were not entirely clear. Several mechanisms have been described as potential regulators of Dicer such as Dicer monoallelic loss [56, 57], downregulated by the transcription factors MITF and TAp63 and miR-103/107 [58, 59]. Furthermore, the latest data illustrated that Dicer expression could also be inhibited by hypoxia through an epigenetic regulation [60].

In our meta-analysis, there seemed to exist some definite associations between low Dicer expression level and poor prognosis results. This trend was extremely obvious in ovarian cancer, ENT tumor, hematologic malignancy and neuroblastoma subgroups. No obvious heterogeneity existed in the four types of tumors that meant the study results in certain cancers were quite consistent, although there were just two trials included in neuroblastoma subgroup. This consequence corresponded to the published mechanisms and we assumed that downregulation of Dicer might promote tumor progression by affecting maturity of some tumor specific suppressing miRNAs.

Despite miRNAs, Dicer is also important for processing other small noncoding RNAs (ncRNAs) which are important for the repair of damaged DNA, so that tumors with normal level of Dicer are able to repair DNA damage caused by oncogenic stress. Partial deletion of Dicer could result in increased but sublethal levels of DNA damage that actually promotes tumorigenicity. In contrast, tumors completely lacking Dicer would accumulate significant DNA damage resulting cell death [61]. This maybe the reason for the inconsistence of the study about breast cancer [14]. Since only in this study, the researcher divide the tissues into Dicer negative and Dicer positive groups which may lead to diametrically opposed result from grouping as low Dicer level and high Dicer level. In the three studies about colorectal cancer, only one study revealed negative result [12]. We compared different aspect of these studies and found out that there was not retrieval step in Faber's study which may affect the result of IHC.

To the best of our knowledge, this was the first study on Dicer level in the prognosis of diverse types of cancers. However, there were several limitations that should not be overlooked. Firstly, high heterogeneities existed in the overall analysis of both OS and PFS, which may be consequence of diversity in tumor histological types since the heterogeneities decreased obviously in subgroup analysis. Study qualities especially experiment method designs may be another main source of heterogeneity. Although we have strictly uniform the inclusion criteria, the type and dilution proportion of antibody as well as cut-off values in both IHC and RT-PCR method were not unified. Besides, sampling error should also not be ignored. Another limitation is that the researches on the role of Dicer in cancer prognosis were not comprehensive since researchers just began to fix their eyes on Dicer in recent 10 years. Only data for prognoses in limiting types of tumors was published but the studies on these aspects were still not enough. And the sample size of each study ranges from 42 to 1,144 indicating that the results of some studies with small sample size were not accurate. What's more, some studies did not provide HR values in the articles directly, so we extract data from the survival curves, which may be less reliable than the HRs and 95% CIs that given directly in the papers.

Thus, further studies were needed to illustrate the complicated regulation network of Dicer in cancer progression, and more homogeneous trails with high quality were necessary for confirming the role of Dicer in cancer prognosis and some negative studies. Despite this, our result still has important implications for clinical prediction of cancers. As in some studies, Dicer is not only a potential clinical detection index but also a promising factor for utilization of personalized novel RNAi-based therapeutics in patients due to its function in RNA interference process [31, 62].

In conclusion, our systematic review and meta-analysis revealed that low Dicer expression level was a significant predictor of poor prognosis in cancer patients especially in ovarian cancer, hematological malignancy, ENT tumor, and neuroblastoma. With more high quality homogeneous studies detecting the expression level of Dicer and the role of Dicer in prognosis of more types of tumors in the future, the predicting role of Dicer will be further confirmed. Until then, Dicer may become a significant clinical parameter in cancer processes and elevating Dicer level may be a new therapy for cancer patients.

## MATERIALS AND METHODS

### Publication searches and study selection

This study began with a comprehensive search of PubMed, Embase, China National Knowledge Infrastructure (CNKI) and WanFang Database online using the Medical Subject Headings (MeSH) combined with keywords search as follows: ‘neoplasm’, ‘cancer’, ‘tumor’, ‘carcinoma’ and ‘dicer’, ‘dicer1’, ‘Ribonuclease III’ and ‘prognos*’, ‘surviv*’, ‘outcome’. All the studies should be published in the form of full article either in English or Chinese language. Searching references listed in reviews and all of the preliminary selected studies were done as complement for this search strategy. The criterions for inclusion included: analyzed the prognosis result in cancer patients according to Dicer lever (accessed dicer lever through immunochemistry (IHC) or accessed mRNA level using RT-PCR). Overall survival (OS) or progression-free survival (PFS) or both was chosen as outcome, and disease free survival (DFS) was regarded to be the same as PFS. After a rigorous screening, detail information of the candidate studies was collected. When the same patient cohort was reported in different publications, only the latest publications or the one with the most complete information was included, in order to avoid the overlap between patient populations.

### Data extraction and methodological assessment

We extracted data of the authors, years of studies and publications, patients' resources, population size, tumor types, stage, study methods (including laboratory methods, cut off values), HR values with 95% confidence intervals (CIs) and treatment from the candidate papers. Three investigators abstracted these data independently and compared the result later, disagreements were discussed to reach consensus by at least two investigators to avoid bias. To assess the methodology, three investigators read all articles independently and scored them according the European Lung Cancer Working Party (ELCWP) scoring scale [63], with some proper modifications (Score scale in Supplementary File). The scores were compared, and a consensus value for each item was defined by at least two investigators. The score evaluating a number of aspects of methodology were grouped into four main categories as follows: design, laboratory methods, generalizability of results and the analysis of the study data. Each category had a maximum score of 10 points, making the theoretical total maximum score of 40 points. The final scores were expressed as percentages ranging from 0 to 100%, with higher values reflecting better methodological quality.

### Statistical methods

We chose HR and 95% CI to evaluate prognostic values. Dicer levels were extracted from articles as high and low dicer level, and we regarded dicer positive as high dicer level and dicer negative as low dicer level. The statistical test comparing low dicer level with high dicer level was considered as significant if *P* < 0.05. For each article, HR and its variance were extracted or evaluated from the information given in the publications. The most accurate method was to extract HR values and 95% CI directly from the published results, or calculate them using the parameters provided in the articles for univariate analysis: the confidence interval (CI) for the HR, the log-rank statistic, its P-value or the O-E statistic (difference between numbers of observed and expected events). If HRs of both univariate and multivariate Cox regression analyses were reported in the articles, only results of univariate cox regression were included in the final analysis. If these information were not available, we counted the total number of events, the number of patients at risk in each group and evaluated the log-rank statistic or its *P*-value, allowing for the calculation of an approximation of the HR estimate. For those articles in which the only useful data was displayed in form of survival curves, we evaluated the outcome values from the graphic information following the method proposed by Tierney [64]. In briefly, we extracted survival rates from survival curves at specific time points using Engauge Digitizer and calculated HR value and 95% CI. In addition, this method was performed by three investigators to reduce imprecision. When low dicer level was reported as a factor of poor outcome for survival, the study was defined as ‘positive’, other results were all defined as ‘negative’.

In this meta-analysis, according to the differences in outcome measurement, analyses were divided into two groups: OS and PFS groups. Subgroup analyses were performed in each group with laboratory method as the variable. If the articles included outcomes of both PCR and IHC method, only the data of IHC method was included. Due to the diversity of tumor types, heterogeneity between studies cannot be neglected, therefore random-effect model was chosen to calculate the overall HR value of all articles using the combination method provided by Yusuf S and Peto R et al [65]. Boxes in the forest plots represent the HR point estimate with horizontal lines indicating the 95% CI, the size of the box is proportional to the number of patients included in each study. Diamond at the bottom of the plot represents the overall HR value. Heterogeneity between studies is assessed by Q-statistic with *P* < 0.10 to be statistically significant. I^2^ value was quantified using the inconsistency index statistic to describe the percentage of variation across studies that are due to heterogeneity rather than chance and classified as no (I^2^ = 0), low (I^2^ < 25%), moderate (I^2^ = 25-50%) and high (I^2^ = 50-100%). Meta regression was performed to assess the probable source of heterogeneity. Sensitivity analysis was applied to evaluate the influence of each article on the stability of the combine result by calculating the pooled HRs in the absence of removing each study. By convention, HR > 1 implied poor survival for the group of low Dicer status. *P* values in all analyses were two sides, with *P* < 0.05 considered to be statistically significantly. Publication bias was investigated using funnel plot and test with Begg's and Egger's test. All analyses were performed with STATA 12.0.

## SUPPLEMENTARY MATERIALS TABLES


