# Supplementary material for: Role of Dicer as a prognostic predictor for survival in cancer patients: a systematic review with a meta-analysis
Source: Oncotarget. 2016 Sep 21;7(45):72672–84. doi: 10.18632/oncotarget.12183 (PMC5341936; doi:10.18632/oncotarget.12183)
Supplement: Supplementary file 1 [file oncotarget-07-72672-s001.doc]

**Role of Dicer as a Prognostic Predictor for Survival in Cancer Patients: A Systematic Review with a Meta-Analysis**

Shan Wanying＃, Sun Chaoyang＃, Zhou Bo＃, Guo Ensong, Lu Hao, Xia Meng, Li Kezhen, Weng Danhui, Lin Xingguang, Meng Li, Ma Ding*, Chen Gang*

**Table 1.Characteristics of Studies Included in Meta-Analysis**

| **First Author** | **Country** | **Tumor Type** | **No. of Case/**  **Control** | **Laboratory Methods** | **Prognostic Result**  **(PFS/ OS)** | **Follow-up**  **(months)**  **mean**  **(start-end)** | **Percent of Low Dicer Level(%)** | **Survival**  **Result** | **Global**  **Score (%)** |
| --- | --- | --- | --- | --- | --- | --- | --- | --- | --- |
| Tchernitsa(2010) | Germany | gastric cancer | 48/142 | IHC | OS | 19.6 | 25.3 | negative | 78.75 |
| Yang(2012) | China | cholangiocarcinoma | 31//9 | IHC | both* | 26.3 (8-48) | 77.5 | positive | 73.75 |
| Papachristou(2012) | USA | soft tissue sarcomas | 35/32 | IHC | OS | 39.16 (3–114) | 52.2 | negative | 75 |
| Caffrey(2013) | Ireland | breast cancer | 301/145 | IHC | both | 48 (1-177) | 67.5 | negative | 81.25 |
| Faber(2011) | Germany | colorectal cancer | 141/96 | IHC | both | 64.8 | 59.5 | negative | 81.25 |
| Diaz-Garcia(2013) | Spain | lung cancer | 76/39 | IHC | OS | 37(1-154) | 66.1 | negative | 86.25 |
| Flavin(2008) | Ireland | ovarian cancer | 17/49 | IHC | both | (0-133) | 26 | negative | 66.25 |
| Khoshnaw(2012) | UK | breast cancer | 597/547 | IHC | PFS | (0-60) | 52.2 | positive | 90 |
| Kawahara(2014） | Japan | oral squamous cell carcinoma | 30/31 | IHC | both | (0-60) | 49 | positive | 90 |
| Shu（2012） | China | gallbladder adenocarcinoma | 36/31 | IHC | OS | (4-18) | 54 | positive | 66 |
| Merritt（2008） | USA | ovarian cancer | 66/45 | IHC | OS | - | 59.5 | positive | 80 |
| Wang（2010） | China | ovarian cancer | 82/19 | IHC | OS | 60 | 81.2 | positive | 73.75 |
| Valencak（2011） | Austria | cutaneous T cell lymphomas | 30/20 | IHC | DFS | 74(1-271) | 60 | negative | 93.75 |
| Faggad(2012） | Germany | colorectal cancer | 65/266 | IHC | OS | 11.1(0.03-118.1) | 19.6 | positive | 88.75 |
| Guo（2012） | China | nasopharyngeal carcinoma | 73/73 | IHC | both | 51(3–96) | 61.2 | positive | 76.25 |
| Grelier（2009） | France | breast cancer | 45/43 | IHC | PFS | (0-180) | 51 | positive | 72.5 |
| Faggad（2010） | Germany | ovarian cancer | 33/107 | IHC | OS | 39(2-107) | 23.6 | positive | 88.75 |
| Liu（2013） | USA | nasopharyngeal carcinoma | 170/106 | IHC | both | 63.6(5.2–91.9) | 61.6 | positive | 88.75 |
| Akahane(2012) | Japan | colorectal cancer | OS:152/108 PFS:109/87 | RT-PCR | both | 45(24-70) | OS:58.5 PFS:55.6 | positive | 83.75 |
| Zighelboim(2011) | USA | endometrial cancer | 84/85 | RT-PCR | both | 59.2(0.7-162.2 ) | 49.7 | positive | 75 |
| Sarasquete(2011) | Spain | symptomatic multiple myeloma | 49/36 | RT-PCR | PFS | - | 57.6 | positive | 50 |
| Kitagawa(2013) | Japan | hepatocellular carcinoma | 47/47 | RT-PCR | PFS | - | 50 | positive | 55 |
| Dedes(2011) | USA | breast cancer | 92/107 | RT-PCR | both | 67(0.5-125） | 46 | negative | 81.25 |
| Bai(2014) | China | acute monocytic leukemia | 15/27 | RT-PCR | OS | 14.6(1-36) | 50 | negative | 58.75 |
| Zhu(2012) | China | chronic lymphocytic leukemia | total 165 | RT-PCR | both | 46 (2-180) | - | positive | 81.25 |
| Zhao(2014) | China | gastrointestinal diffuse large B-cell lymphoma | total 62 | RT-PCR | both | - | 50 | positive | 72.5 |
| Karube(2005) | Japan | lung cancer | 11/56 | RT-PCR | OS | - | 16.4 | positive | 63.75 |
| Lin(2010) | Taiwan | neuroblastoma | 21/44 | RT-PCR | both | DFS:64 OS:73 | 32.4 | positive | 72.5 |
| Feinberg-  Gorenshtein(2013) | Israel | neuroblastoma | 23/24 | RT-PCR | PFS | 110(4-289) | 48.9 | positive | 56.25 |
| Sugito(2006) | Japan | esophageal cancer | 48/25 | RT-PCR | both | (0-50) | 65.8 | negative | 70 |
| He(2014) | China | cervical cancer | 52/50 | RT-PCR IHC | both | (0-120) | 51 | positive | 88.75 |
| Lonvik (2014) | Norway | Non-small-cell lung cancer | 241/80 | IHC | DFS | 86(48-216) | 75.1 | positive | 87.5 |
| Zhang (2015) | China | Bladder carcinoma | 30/70 | RT-PCR | DFS | 35(3-100) | 30 | positive | 85.0 |
| Xu (2015) | China | nasopharyngeal carcinoma | 170/106 | IHC | OS | 63.6(5.2-91.9) | 38 | positive | 80.5 |

*both: the study provided results of both PFS and OS. IHC: Immunohistochemistry. RT-PCR: Real Time Polymerase Chain Reaction. Total number: the study didn’t provide detail number of control and case group.

**Table 2.Characteristics of Studies of Patients with Low Dicer Expression Measured by IHC.**

| **First Author** | **Country** | **Tumor Type** | **No. of Case/Control** | **Retrieval** | **Clone** | **Dilution** | **Readers** | **Double Blind** | **Cut-off** | **Low Dicer%** | **Survival Result** |
| --- | --- | --- | --- | --- | --- | --- | --- | --- | --- | --- | --- |
| Tchernitsa(2010) | Germany | gastric cancer | 48/142 | pressure cooker | polyclonal anti-Dicer antibody | 1:100 | 1 | yes | IRS=1 | 25.3 | negative |
| Yang(2012) | China | cholangiocarcinoma | 31/9 | no | monoclonal anti-Dicer antibody | 1:100 | 2 | yes | IRS=4 | 77.5 | positive |
| Papachristou(2012) | USA | soft tissue sarcomas | 35/32 | no | anti-Dicer antibody ab14601 | 1：50 | 2 | yes | IRS=1 | 52.2 | negative |
| He(2014) | China | cervical cancer | 52/50 |  | mouse monoclonal antibody | 1:400 | 2 | yes | IRS=2 | 51 | positive |
| Caffrey(2013) | Ireland | breast cancer | 301/145 | microwave | rabbit polyclonal antibody, (13D6R) mouse monoclonal antibody, ( 13D6) | 1:2500 | 2 | yes | IRS=0 | 67.5 | negative |
| Faber(2011) | Germany | colorectal cancer | 141/96 | no | anti-Dicer1 antibody | 1:75 | 1 | yes | IRS=1 | 59.5 | negative |
| Diaz-Garcia(2013) | Spain | non-small cell lung cancer | 76/39 | Leica Bond III automated system | antibodies against DICER1 | 1:200 | 2 | yes | IRS=2 | 66.1 | negative |
| Flavin(2008) | Ireland | ovarian cancer | 17/49 | microwave | mouse monoclonal anti-Dicer antibody (13D6) | 1:50 | 2 | yes | IRS=1.6 | 25.8 | negative |
| Khoshnaw(2012) | UK | breast cancer | 597/547 | microwave | mouse monoclonal anti-Dicer antibody (13D6） | 1:300 | 2 | yes | H-score =157 | 52.2 | positive |
| Kawahara(2014） | Japan | oral squamous cell carcinoma | 30/31 | autoclave | rabbit polyclonal anti-Dicer antibody | 1:600 | 3 | yes | IRS=2 | 49.2 | positive |
| Shu（2012） | China | gallbladder adenocarcinoma | 36/31 | no | rabbit anti-Dicer antibody | 1:100 | - | - | positive cells =25% | 53.7 | positive |
| Merritt（2008） | USA | ovarian cancer | 66/45 | pressure cooker | anti-Dicer antibody | 1:100 | 2 | yes | score of IHC=100 | 59.5 | positive |
| Wang（2010） | China | ovarian cancer | 82/19 | pressure cooker | mouse monnclonal anti-Dicer antibody | 1:200 | - | - | IRS=7 | 81.2 | positive |
| Guo（2012） | China | nasopharyngeal carcinoma | 73/73 | not mention | rabbit anti-Dicer antibody | 1:50 | - | - | T test= 78.8% | 50 | positive |
| Valencak（2011） | Austria | primary cutaneous T cell lymphomas | 30/20 | autoclave | mouse monoclonal anti-Dicer antibody (13D6) | 1:50 | 2 | yes | IRS=2 | 60 | negative |
| Faggad（2012） | Germany | colorectal cancer | 65/266 | pressure cooker | Mouse monoclonal anti-Dicer antibody(13D6) | 1:600 | 2 | yes | IRS=0 | 19.6 | positive |
| Grelier（2009） | France | breast cancer | 45/43 | water bath | mouse monoclonal anti-Dicer antibody (13D6) | 1:150 | 2 | yes | IRS=8 | 51 | positive |
| Faggad（2010） | Germany | ovarian cancer | 33/107 | pressure cooker | mouse monoclonal anti-Dicer antibody | 1:300 | - | yes | IRS=6 | 23.6 | positive |
| Liu（2013） | USA | nasopharyngeal carcinoma | 170/106 | microwave | mouse monoclonal anti-Dicer1 antibody | 1:200 | 2 | yes | IRS=4 | 61.6 | positive |
| Lonvik (2014) | Norway | Non-small-cell lung cancer | 241/80 | microwave | Mouse monoclonal anti-Dicer antibody (13D6) | 1:20 | 2 | yes | IRS=2 | 87.5 | positive |
| Xu (2015) | China | nasopharyngeal carcinoma | 170/106 | microwave | Mouse monoclonal anti-Dicer antibody (13D6) | 1:200 | 2 | yes | IRS=4 | 80.5 | Positive |

IRS: immunoreactive score. Low Dicer%: the percent of patients with low Dicer expression.

**Table 3.Characteristics of Studies of Patients with Low Dicer Expression Measured by RT-PCR.**

| **First Author** | **Country** | **Tumor Type** | **No. of**  **Case/Control** | **Prognostic Result**  **PFS/ OS** | **Percent of Low**  **Dicer level(%)** | **Survival**  **Result** | **Mean Global**  **Score (%)** |
| --- | --- | --- | --- | --- | --- | --- | --- |
| Zighelboim(2011) | USA | primary endometrial cancer | 84/85 | both* | 49.7 | positive | 75 |
| Sarasquete(2011) | Spain | symptomatic multiple myeloma | 49/36 | PFS | 57.6 | positive | 50 |
| Kitagawa(2013) | Japan | hepatocellular carcinoma | 47/47 | PFS | 50 | positive | 55 |
| Dedes(2011) | USA | breast cancer | 92/107 | both | 46 | negative | 81.25 |
| Bai(2014) | China | acute monocytic leukemia | 15/27 | OS | 50 | negative | 58.75 |
| Zhu(2012) | China | chronic lymphocytic leukemia | total 165 | both | - | positive | 81.25 |
| Karube(2005) | Japan | lung cancer | 11/56 | OS | 16.4 | positive | 63.75 |
| Sugito(2006) | Japan | esophageal cancer | 48/25 | both | 65.8 | negative | 70 |
| He(2014) | China | cervical cancer | 52/50 | both | 51 | positive | 88.75 |
| Feinberg-Gorenshtein(2013) | Israel | neuroblastoma | 23/24 | PFS | 48.9 | positive | 56.25 |
| Zhao(2014) | China | primary gastrointestinal diffuse  large B-cell lymphoma | total 62 | both | 50 | positive | 72.5 |
| Lin(2010) | Taiwan | neuroblastoma | 21/44 | both | 32.4 | positive | 72.5 |
| Zhang(2015) | China | Bladder carcinoma | 30/70 | DFS | 30 | positive | 85.0 |
| Akahane(2012) | Japan | colorectal cancer | OS:152/108 PFS:109/87 | both | OS:58.5 PFS:55.6 | positive | 83.75 |

*both: the study provided results of both PFS and OS. Total number: the study didn’t provide detail number of control and case group.

Figure 1
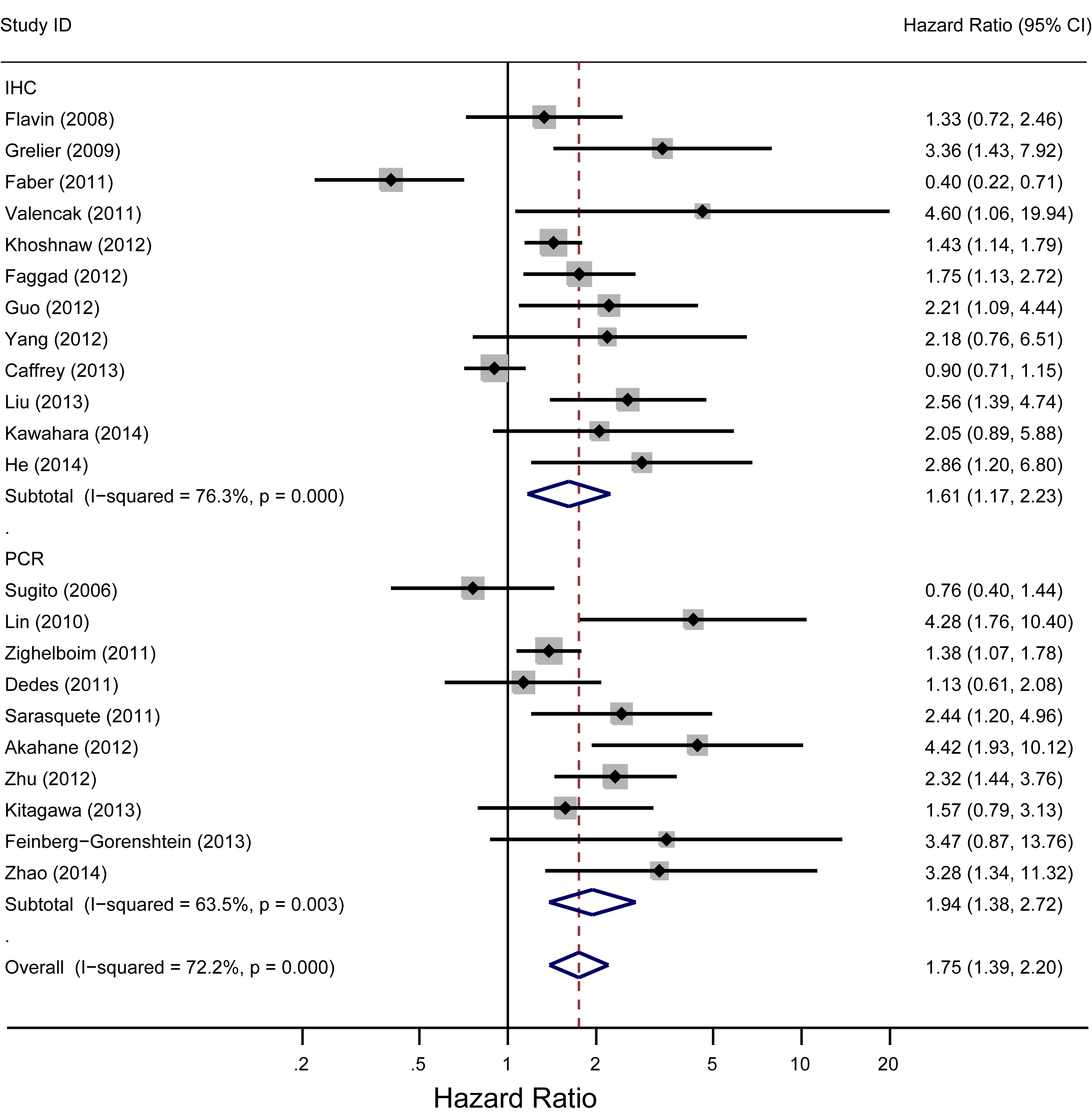


Summary hazard ratios and 95% confidence intervals (CIs) of cancer patients in PFS group after excluding the two studies that impact on the whole stability of PFS group. Horizontal lines represent 95% CIs; diamonds represent summary estimates with corresponding 95% CIs. A random-effects model was used for analysis. Test for heterogeneity: I^2^ =72.2%, P=0.000.

**The** **quality score for methodology modified according to the European Lung Cancer Working Party (ELCWP) scoring scale ^[1]^.**

Except when specified, the attributed value per item is 2 points if it is clearly defined in the article, 1 point if its description is incomplete or unclear and 0 point if it is not defined or is inadequate**.**

**Scientific design**

1. Study objective definition.
2. Study design: prospective (2 points); retrospective (1 point); not defined (0 point).
3. Outcome definition.
4. Statistical considerations: fully reported with a preliminary assessment of the patient/sample number to be included and/or analysed (2 points); patient/sample number to be included and/or analysed justified by the number of studied variables (minimum 10 patients per variable) (1 point); not defined (0 point).
5. Statistical methods and tests description.

**Laboratory methodology**

1. Blinding in the biological assays performance: double-blind (2 points); simple-blind (1 point); unblinded or not defined (0 point).
2. Tested factor description: DNA (types of exons analysed), messenger RNA (complete or partial with description of the primers used), protein (nuclear, cytoplasmic or extracted from cellular components), antibodies (type of tissue or liquid sampled).
3. Tissue sample conservation: either fresh tissue or conservation requiring freezing at ≤−80°C in presence of an anti-RNAase for RNA or freezing at ≤−20°C for DNA, protein and serum, or fixation in formol, alcohol or paraffin.
4. Description of the revelation test procedure of the biological factor: PCR with mention of primers, polymerase type, general reaction conditions (concentration of the various reagents, cycle number, duration and temperature of the various steps); IHC with the first antibody type and clone identification, second antibody type, reaction characteristics (antibodies concentration, duration and temperature of incubation), colouration method (peroxydase, alkaline phosphatase or chromagenic method), epitope unmasking method in case of fixed tissue, endogenous peroxydase activity inhibition method if the colouration method requires peroxidase.
5. Description of the negative and positive control procedures. Definition of the positive or negative results.

**Generalizability**

1. Patient selection criteria, including histological type, disease stage and treatment.
2. Patients' characteristics, including histology type, disease stage and treatment.
3. Treatment description.
4. Source of samples.
5. Number of unassessable samples with exclusion causes.

**Results analysis**

1. Follow-up description, including the number of events.
2. Statistics description
3. Survival analysis according to the biological marker.
4. Univariate analysis of the prognostic factors for survival: report of the hazard ratio with the confidence interval (2 points); results without evaluation of the hazard ratio and its confidence interval (1 point); not reported or inadequate (0 point).
5. Multivariate analysis of the prognostic factors for survival: report of the hazard ratio with the confidence interval (2 points); results without evaluation of the hazard ratio and its confidence interval (1 point); not reported or inadequate (0 point)

**Reference**

**[1]** Steels E, Paesmans M, Berghmans T, Branle F, Lemaitre F, et al. (2001) Role of p53 as a prognostic factor for survival in lung cancer: a systematic review of the literature with a meta-analysis. Eur Respir J 18: 705-719.
